# Spatial Distribution of Human *Schistosoma japonicum* Infections in the Dongting Lake Region, China

**DOI:** 10.1371/journal.pone.0006947

**Published:** 2009-09-14

**Authors:** Giovanna Raso, Yuesheng Li, Zhengyuan Zhao, Julie Balen, Gail M. Williams, Donald P. McManus

**Affiliations:** 1 Département Environnement et Santé, Centre Suisse de Recherches Scientifiques, Abidjan, Côte d'Ivoire; 2 Swiss Tropical Institute, Basel, Switzerland; 3 Molecular Parasitology Laboratory, Queensland Institute of Medical Research, Brisbane, Queensland, Australia; 4 Hunan Institute of Parasitic Diseases, WHO Collaborating Centre for Research and Control on Schistosomiasis in Lake Region, Yueyang, Hunan Province, People's Republic of China; 5 School of Public Health, Central South University, Changsha, Hunan Province, People's Republic of China; 6 School of Population Health, The University of Queensland, Brisbane, Queensland, Australia; 7 Centre for Non-Traditional Security Studies, S. Rajaratnam School of International Relations, Nanyang Technological University, Singapore, Singapore; New Mexico State University, United States of America

## Abstract

**Background:**

The aim of this study was to spatially model the effect of demographic, reservoir hosts and environmental factors on human *Schistosoma japonicum* infection prevalence in the Dongting Lake area of Hunan Province, China and to determine the potential of each indicator in targeting schistosomiasis control.

**Methodology/Principal Findings:**

Cross-sectional serological, coprological and demographic data were obtained from the 2004 nationwide periodic epidemiologic survey for Hunan Province. Environmental data were downloaded from the USGS EROS data centre. Bayesian geostatistical models were employed for spatial analysis of the infection prevalence among study participants. A total of 47,139 participants from 47 administrative villages were selected. Age, sex and occupation of residents and the presence of infected buffaloes and environmental factors, i.e. NDVI, distance to the lake and endemic type of setting, were significantly associated with *S. japonicum* infection prevalence. After taking into account spatial correlation, however, only demographic factors (age, sex and occupation) and the presence of infected buffaloes remained significant indicators.

**Conclusions/Significance:**

Long established demographic factors, as well presence of host reservoirs rather than environmental factors are driving human transmission. Findings of this work can be used for epidemiologic surveillance and for the future planning of interventions in the Dongting Lake area of Hunan Province.

## Introduction

In China, the blood fluke *Schistosoma japonicum* is the causative agent of schistosomiasis, a chronic and debilitating disease, which occurs mainly in the marsh and lake regions in the south, covering a vast area of five provinces (Anhui, Hubei, Hunan, Jiangsu and Jiangxi). Further endemic foci are also known in the mountainous regions of Sichuan and Yunnan [1], [2]. In spite of remarkable control efforts, schistosomiasis remains a public health problem in China [3], [4]. Demographic and ecological transformations, resettlement of communities due to large water management projects [5], market-based reforms of the health sector [6], and the end of the World Bank loan project on schistosomiasis control [7] have hindered control progress.

Unlike other schistosomiasis forms in humans, schistosomiasis japonica is a zoonotic disease, which makes control efforts more difficult. At present, there are over 40 known mammalian species that are capable of acting as a reservoir for the infection [8]. Water buffaloes are the major host reservoir for transmission to humans in the lake regions, accounting for up to 80% of transmission [9], [10]. The schistosome life cycle includes an amphibious freshwater snail, *Oncomelania hupensis* that releases infectious free swimming larval forms of the parasite (cercariae), which in turn can penetrate the host skin. Four subspecies of the snail occur in mainland China, i.e. *O. hupensis guagxiensis*, *O. hupensis hupensis*, *O. hupensis robertsoni* and *O. hupensis tangi* [11]. Current estimates suggest that approximately 726,000 people and over 100,000 cattle and buffaloes are infected with *S. japonicum* in China [8].

An important feature of schistosomiasis is its focal distribution. Consequently, there is a need for rapid assessment procedures to identify communities at highest risk of the disease. The development of geographical information system (GIS) and remote sensing technologies and their application to health issues in general, and tropical infectious diseases in particular, has a short history of approximately 10–15 years [12]–[16]. Nevertheless, these techniques have become important tools in China's national schistosomiasis control programme. Predictions on infection risk were mainly made by the application of normalized difference vegetation index (NDVI) or land surface temperature (LST) to predict intermediate host snail habitats. Significant advances have been made with Bayesian approaches, which allow flexible modeling and inference, and provide computational advantages over frequentist analyses via the implementation of Markov chain Monte Carlo (MCMC) methods [17]. Bayesian approaches allow spatial dependence to be modeled in a hierarchical fashion, by introducing area or site-specific random effects with conditional autoregressive [18] or Gaussian random field prior specifications [19]. These advances have greatly improved spatial data analysis, including disease risk mapping and prediction at non-sampled locations. Recent studies in China utilized Bayesian geo-statistical modelling to investigate spatial and spatio-temporal correlations of *S. japonicum* prevalence data and to assess how environmental factors affect these correlations [20]–[22].

We employed individual-level cross-sectional epidemiologic data from the 2004 periodic epidemiologic survey carried out by the Chinese Ministry of Health in the Dongting Lake area of Hunan province to model the effect of demographic, reservoir hosts and environmental factors on human *S. japonicum* infection prevalence using Bayesian geostatistical models and to discuss the potential of each indicator in targeting schistosomiasis control.

## Materials and Methods

### Study area and environmental data

The study was carried out in the framework of a nationwide cross-sectional epidemiologic survey by the Chinese Ministry of Health in October/November 2004. The survey was conducted in 47 administrative villages in the Dongting Lake region in Hunan province where schistosomiasis is endemic. The study area and sampling procedure have been presented in detail [23].

In China, each administrative village comprises several natural villages within a discrete area. Geographic coordinates were collected at the centre of each administrative village (i.e. at the centre of several natural villages) using a hand-held global positioning system (GPS; Thales Navigation, Santa Clara, CA, USA).

Rivers and lake boundaries were available through digitized maps. The straight distance in kilometers between an administrative village and the Dongting Lake was calculated using ArcGIS v.9 (ESRI, Redlands, CA, USA). Monthly normalized difference vegetation index (NDVI) were downloaded at 1×1 km spatial resolution for the year 2004 from Moderate Resolution Imaging Spectroradiometer (MODIS) from the USGS EROS Data Centre. Individual scenes were mosaicked and thereafter resized in ENVI 4.0 (Research Systems, Inc., Boulder, USA). Values were extracted for each sampled administrative village. Information on type of endemic setting for each sampled location was extracted from available maps.

### Serological and coprological examinations

Briefly, serum was extracted from 2 ml venous blood samples taken from each participant, and examined by the indirect enzyme linked immunosorbent assay (ELISA) for the presence of anti-soluble egg antigen (SEA) IgG antibodies [24]. Thereafter, all SEA-ELISA positive individuals were invited to provide a stool specimen. Three Kato-Katz thick smears [25] were prepared from each specimen and examined under a light microscope for the presence of *S. japonicum* eggs by experienced laboratory technicians.

The miracidial hatching test was carried out on single stool specimens taken from buffaloes, to identify *S. japonicum* positive animals [26].

### Ethical approval, consent and anthelmintic treatment

This study was organized by the National Institute of Parasitic Diseases and approved by the Chinese Center of Disease Control and the Chinese Ministry of Health Beijing, China. The buffalo component of the study was conducted following the animal husbandry guidelines described in the handbook of schistosomiasis control released by the Ministry of Public Health of the People's Republic of China [27]. Written informed consent was obtained from each individual by the head of each participating administrative village and Chinese Ministry of Health officials before commencement of the study. For children under the age of 15 years, written informed consent was obtained from their parent/legal guardian. Verbal informed consent was obtained from the domestic animal owners by Chinese Ministry of Health officials. ELISA-positive individuals, apart from pregnant women, were treated with praziquantel (single oral dose of 40 mg/kg bodyweight). All *S. japonicum*-positive buffaloes were also treated with single oral doses of praziquantel at 25 mg/kg.

### Data management and statistical analysis

The data were double-entered into a FoxPro database (version 6.0) and cross-checked. Participants were classified into six age categories (0–10 years, 11–20 years, 21–30 years, 31–40 years, 41–50 years and >50 years). Environmental covariates were standardized with a mean of 0 and a standard deviation of 1.

All demographic, reservoir related and environmental covariates were fitted into bivariate regressions on the infection status variable (based on ELISA and Kato-Katz) in STATA v. 9.0 (Stata Corporation, College Station, TX, USA). Covariates with a significance level<0.15 were built into multivariate spatial models for *S. japonicum* infection based on ELISA and Kato-Katz examinations using WinBUGS v.1.4 (Imperial College & Medical Research Council, London, UK). Spatial heterogeneity was taken into account by introducing location-specific random effects, which model a latent spatial process. Significance of a covariate in the spatial models was assessed by inspecting the credible intervals of the estimated coefficients/odds ratios.

### Model specification

Let 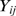 and 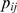 be the infection status and the probability of infection with *S. japonicum* of participant *j* in village *i*. We assumed that 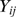 arise from a Bernoulli distribution, 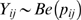. Covariates 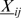 and village-specific random effects 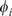
were modelled on the 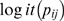, that is 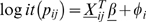 where 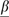 is the vector of regression coefficients. Spatial correlation was introduced on the 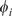
's by assuming that 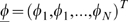 has a Multivariate Normal distribution 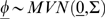 with variance-covariance matrix 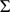. We also assumed an isotropic spatial process where 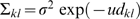, 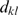
is the Euclidean distance between villages *k* and *l*, 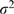
is the geographical variability known as the sill. 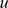 is a smoothing parameter which controls the rate of correlation decay with increasing distance.

Following a Bayesian model specification, we chose vague normal distributions for the 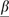 parameters with large variances (i.e. 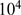), an inverse gamma prior for 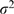 and a uniform prior for 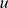. Markov chain Monte Carlo (MCMC) simulation was applied to fit the models. We run a single chain sampler with a burn-in of 2000.

For comparison we fitted 5 models. We first fitted a non spatial logistic regression model with only the constant (Model 1). In a next step we introduced a spatial random effect to the model (Model 2). We then fitted three further spatial models that included demographic covariates (Model 3), demographic and reservoir host covariates (Model 4) and demographic, reservoir host and environmental covariates (Model 5). The deviance information criterion (DIC) was used to compare model performance.

## Results

### Cohort characteristics and infections with S. japonicum

Overall, 47,139 participants from 47 administrative villages were selected for the analyses, namely those with complete serological, coprological and demographic information. There were 3,598 (7.6%) children aged<11 years, 9,039 (19.2%) children and young adults aged 11–20 years, 3,782 (8.0%) participants aged 21–30 years, 10,759 (22.8%) participants aged 31–40 years, 8,746 (18.6%) participants aged 41–50 years and 11,215 (23.8%) were over 50 years. Furthermore, 22,502 (47.7%) participants were male.

**Figure 1:**
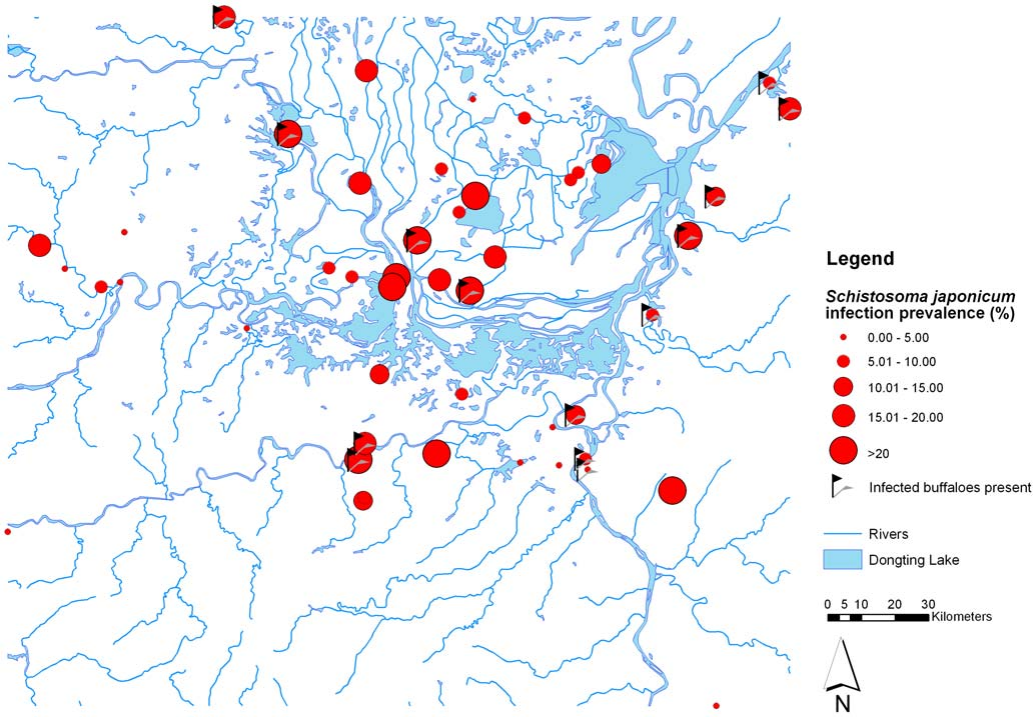
Map of the Dongting Lake area (Hunan Province, China) showing the human *S. japonicum* village prevalence based on ELISA, as well as locations where *S. japonicum* infected buffaloes were found.

**Figure 2:**
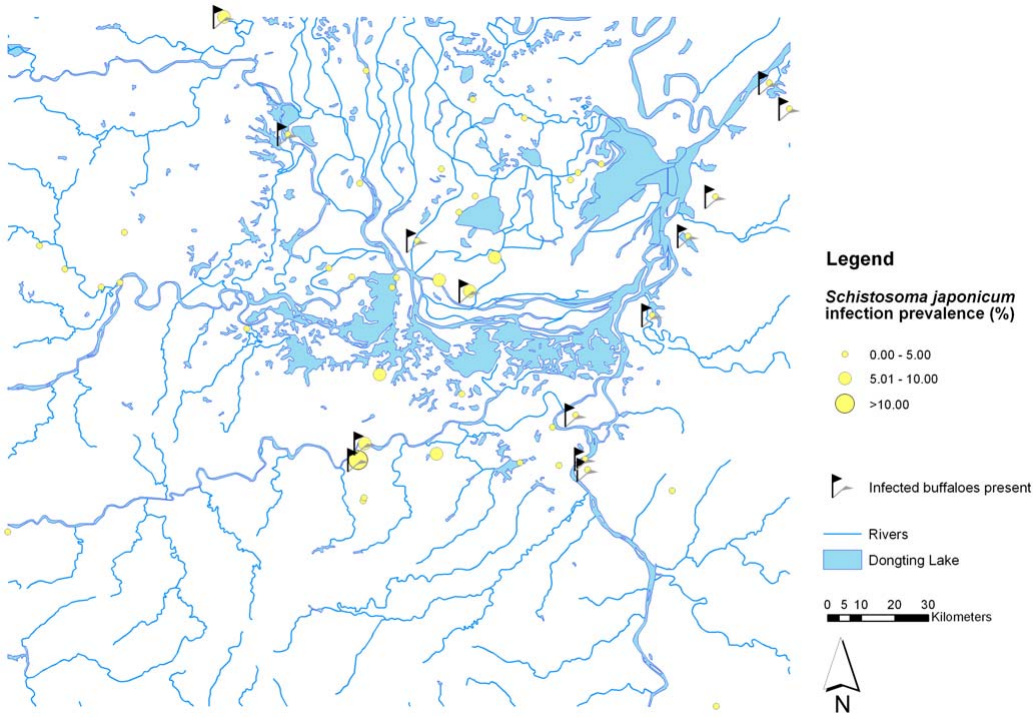
Map of the Dongting Lake area (Hunan Province, China) showing the human *S. japonicum* village prevalence based on repeated Kato-Katz readings, as well as locations where *S. japonicum* infected buffaloes were found.

**Figure 3:**
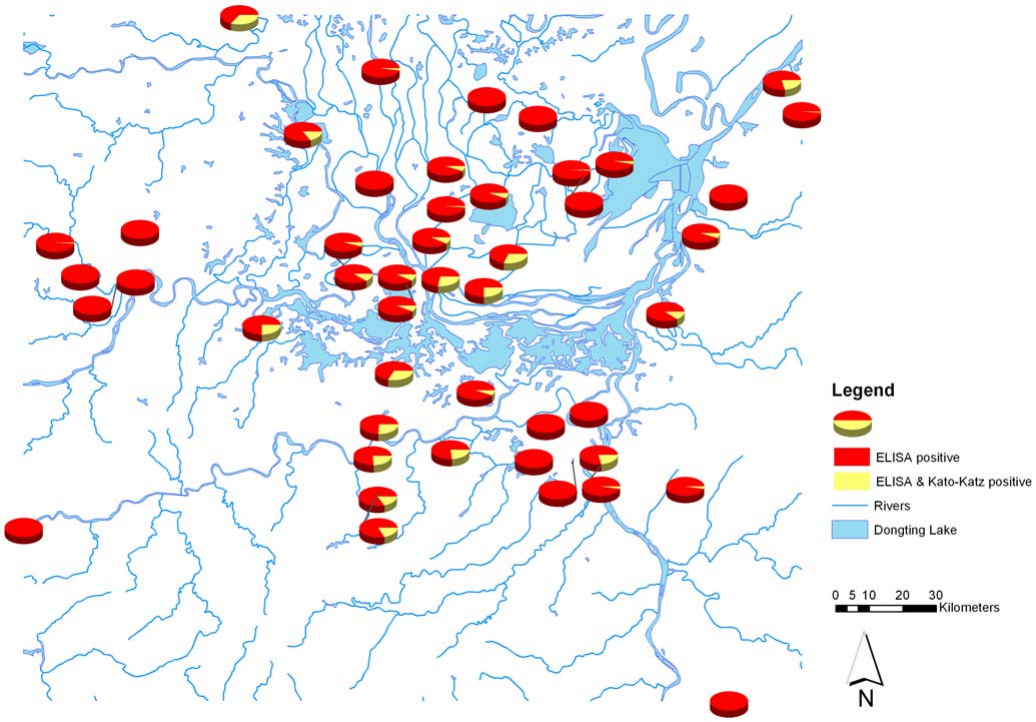
Proportion of study participants that were *S. japonicum* positive only with ELISA compared to those that were *S. japonicum* positive with both ELISA and Kato-Katz thick smear in the Dongting Lake area, Hunan Province, China.

Most participants were engaged in herding, farming and fishing (33,788 participants, 71.7%), followed by students and preschool children (11,482, 24.4%), and civil servants and businessmen (1,282, 2.7%). Infected buffaloes were found in 14 (29.8%) of the 47 administrative villages and the buffalo infection prevalence ranged from 0% to 66.7% at the administrative village level. A total of 5,624 (11.9%) participants tested positive for a *S. japonicum* infection with the ELISA method and the prevalence ranged from 1.2% to 34.9% at administrative village level. As expected, the overall infection prevalence based on Kato-Katz thick smear was lower (874 participants, 1.9%), ranging from 0% to 10.8% among administrative villages. Figure 1 shows the *S. japonicum* infection prevalence based on the ELISA method results at administrative village level, as well as the villages where *S. japonicum* infected buffaloes were found. Similarly, Figure 2 shows the prevalence based on the Kato-Katz thick smear in the study locations. The proportion of study participants that were *S. japonicum* positive only with ELISA compared to those that were *S. japonicum* positive with both ELISA and Kato-Katz thick smear at administrative village level is presented in Figure 3.

### Associations with S. japonicum infection prevalence

Demographic covariates, i.e. age and sex, were positively and significantly associated with the *S. japonicum* infection prevalence based on either ELISA or Kato-Katz thick smear results. Males were more likely to be infected than female participants (ELISA: 15.3% vs. 8.3%; Kato-Katz: 2.7% vs. 0.9%). The ELISA-based infection prevalence for children aged<11 years, children and young adults aged 11–20 years, participants aged 21–30 years, participants aged 31–40 years, participants aged 41–50 years and participants over 50 years was 3.3%, 6.5%, 10.8%, 13%, 15.2% and 15.9%, respectively and for the Kato-Katz-based infection prevalence 0.3%, 0.5%, 1.5%, 2%, 2.7% and 2.8%, respectively. Occupational activities such as herding, fishing and farming increased the risk of an infection with *S. japonicum*; this was found to be significant for both ELISA and Kato-Katz thick smear results. The presence of *S. japonicum* infected buffaloes, including the overall village prevalence of *S. japonicum* infected buffaloes, were also associated with a higher infection risk. Likewise, the NDVI was a significant indicator for *S. japonicum* infection based on either ELISA or Kato-Katz thick smear results. Additionally, people living further away from the lake (only for results based on ELISA) and within a lake embankment setting were at a higher risk of having an *S. japonicum* infection. The result that living further away from the lake was associated with a higher risk of infection is surprising and needs further investigation. Detailed results of the associations between human infection status and demographic, reservoir and environmental factors are presented in Table 1.

**Table 1:** Comparison of bivariate non-spatial models for *S. japonicum* infections based on either ELISA or Kato-Katz results with demographic, reservoir and environmental covariates that were significant at a p-value<0.15.

Covariate group	Covariate	Covariate category	ELISA			Kato-Katz		
			OR^a^	CI^b^	AIC^c^	OR^a^	CI^b^	AIC^c^
Demography								
	Age group (years)							
		0–10	1			1		
		11–20	2.06	1.68, 2.52		1.56	0.83, 2.95	
		21–30	3.59	2.91, 4.43		4.41	2.36, 8.25	
		31–40	4.42	3.65, 5.35		6.01	3.35, 10.76	
		41–50	5.27	4.34, 6.38		8.32	4.65, 14.89	
		>50	5.56	4.60, 6.73	33584	8.52	4.78, 15.19	8442
	Sex							
		Female	1			1		
		Male	1.99	1.88, 2.11	33914	3.12	2.66, 3.65	8474
	Occupation							
		Herdsman, farmer, fisherman or boatman	1			1		
		Preschool or student	0.33	0.30, 0.36		0.17	0.13, 0.23	
		Civil servant or businessman	0.88	0.75, 1.04		0.22	0.11, 0.47	
		Other	0.52	0.39, 0.71	33690	0.56	0.27, 1.13	8456
Reservoir								
	Presence of infected buffaloes in the village		1.76	1.66, 1.86	34104	2.96	2.60, 3.40	8458
	Prevalence of infected buffaloes in the village		1.25	1.22, 1.28	34136	1.35	1.29, 1.41	8571
Environment								
	Mean NDVI		1.20	1.16, 1.24	34337	0.98	0.92, 1.05	8706
	Mean NDVI during spring, summer and fall		1.31	1.26, 1.35	34204	1.16	1.07, 1.25	8691
	Mean NDVI during winter		1.08	1.05, 1.11	34439	0.86	0.81, 0.91	8682
	Distance to Dongting Lake (km)		1.08	1.05, 1.11	34438	1.05	0.99, 1.13	8703
	Endemic types							
		Lake embankment	1			1		
		Inside embankment	0.69	0.61, 0.78		0.24	0.17, 0.33	
		Lake fork	1.02	0.94, 1.12		0.72	0.60, 0.85	
		Grassy lake beach/marshland	0.78	0.70, 0.86		0.16	0.12, 0.21	
		Hills/hilly area	0.52	0.45, 0.61	34292	0.02	0.01, 0.07	8324

a OR: Odds ratio.

b CI: Confidence interval.

c Akaike information criterion. The smaller the AIC the better the model performance.

### Multivariate spatial analysis

Measures of association and the respective credibility intervals of the multivariate analyses are shown in Table 2 for results based on ELISA and in Table 3 for results based on Kato-Katz thick smear. Summarizing the results of the different multivariate models presented in Tables 2 and 3, it appears that only demographic factors (age, sex and occupation) and the presence of infected buffaloes were significant indicators for human infection prevalence after spatial correlation had been taken into account.

**Table 2:** Comparison of 5 different non-spatial and spatial models for *S. japonicum* infections based on ELISA examination showing the importance of including spatial correlation in the analyses as well as the inclusion of the different demographic, reservoir and environmental covariates.

Covariate group	Covariate	Covariate category	Bayesian non-spatial	Bayesian spatial
			Model 1	Model 2	Model 3	Model 4	Model 5
			OR^a^	BCI^b^	OR^a^	BCI^b^	OR^a^	BCI^b^	OR^a^	BCI^b^	OR^a^	BCI^b^
Demography												
	Age group (years)											
		0–10					1		1		1	
		11–20					1.87	1.52, 2.29	1.87	1.51, 2.31	1.89	1.52, 2.31
		21–30					2.29	1.69, 3.09	2.27	1.65, 3.15	2.32	1.68, 3.06
		31–40					2.79	2.09, 3.71	2.77	2.04, 3.78	2.82	2.07, 3.68
		41–50					3.31	2.47, 4.41	3.28	2.42, 4.51	3.35	2.44, 4.37
		>50					3.51	2.62, 4.68	3.49	2.57, 4.77	3.56	2.60, 4.65
	Sex											
		Female					1		1		1	
		Male					2.08	1.96, 2.21	2.08	1.96, 2.21	2.08	1.96, 2.22
	Occupation											
		Herdsman, farmer, fisherman or boatman					1		1		1	
		Preschool or student					0.60	0.48, 0.75	0.60	0.47, 0.75	0.60	0.48, 0.74
		Civil servant or businessman					0.70	0.52, 0.91	0.70	0.52, 0.92	0.71	0.53, 0.93
		Other					0.50	0.35, 0.67	0.50	0.36, 0.67	0.50	0.36, 0.67
Reservoir												
	Presence of infected buffaloes in the village								2.26	1.24, 3.76	2.40	1.32, 4.15
Environment												
	Mean NDVI during spring, summer and fall										0.97	0.95, 1.02
	Distance to Dongting Lake (km)										1.21	0.93, 1.60
	Endemic types											
		Lake embankment									1	
		Inside embankment									1.02	0.42, 2.10
		Lake fork									0.73	0.31, 1.56
		Grassy lake beach/marshland									1.20	0.43, 2.73
		Hills/hilly area									0.76	0.18, 2.11
*u* c					49.74	3.98, 234.10	64.17	5.84, 241.10	51.57	5.38, 234.70	47.92	4.60, 236.30
*σ^2^* ^d^					0.90	0.55, 1.54	0.88	0.56, 1.40	0.78	0.48, 1.30	0.83	0.50, 1.40
DIC^e^			34464		31766		30292		30293		30293	

a OR: odds ratio.

b BCI: Bayesian credible interval.

c *u* is scalar parameter representing the rate of decline of correlation with distance between points.

d *σ^2^* is the estimate of the geographic variability.

e DIC is the measure for the model fit. A smaller DIC indicates a better performance of the model.

**Table 3:** Comparison of 5 different non-spatial and spatial models for *S. japonicum* infections based on Kato-Katz examination showing the importance of including spatial correlation in the analyses as well as the inclusion of the different demographic, reservoir and environmental covariates.

Covariate group	Covariate	Covariate category	Bayesian non-spatial	Bayesian spatial
			Model 1	Model 2	Model 3	Model 4	Model 5
			OR^a^	BCI^b^	OR^a^	BCI^b^	OR^a^	BCI^b^	OR^a^	BCI^b^	OR^a^	BCI^b^
Demography												
	Age group (years)											
		0–10					1		1		1	
		11–20					1.36	0.69, 2.65	1.43	0.70, 2.61	1.47	0.75, 2.68
		21–30					2.44	0.89, 5.84	2.64	0.95, 5.90	2.80	1.08, 6.13
		31–40					3.06	1.16, 7.29	3.31	1.22, 7.31	3.51	1.40, 7.60
		41–50					4.22	1.58, 10.05	4.56	1.69, 10.07	4.85	1.94, 10.55
		>50					4.47	1.67, 10.69	4.82	1.79, 10.50	5.13	2.09, 11.05
	Sex											
		Female					1		1		1	
		Male					3.18	2.70, 3.75	3.18	2.70, 3.74	3.20	2.71, 3.77
	Occupation											
		Herdsman, farmer, fisherman or boatman					1		1		1	
		Preschool or student					0.48	0.23, 0.91	0.50	0.24, 0.93	0.52	0.26, 0.96
		Civil servant or businessman					0.40	0.11, 0.93	0.40	0.12, 0.92	0.41	0.12, 0.94
		Other					0.62	0.25, 0.91	0.63	0.27, 1.17	0.63	0.26, 1.17
Reservoir									1		1	
	Presence of infected buffaloes in the village								5.34	1.14, 16.03	7.04	1.56, 24.23
Environment												
	Mean NDVI during winter										0.74	0.31, 1.69
	Distance to Dongting Lake (km)										1.22	0.55, 2.33
	Endemic types											
		Lake embankment									1	
		Inside embankment									2.15	0.15, 9.76
		Lake fork									0.51	0.03, 2.20
		Grassy lake beach/marshland									13.49	0.43, 75.17
		Hills/hilly area									16.07	0.01, 57.75
*u* c					15.21	2.51, 137.50	19.96	2.64, 185.50	19.47	3.11, 175.40	11.26	1.58, 87.00
*σ^2^* ^d^					6.35	3.08, 13.32	6.50	3.26, 13.22	5.92	2.88, 11.43	7.29	2.98, 18.89
DIC^e^			8705		7149		6652		6652		6649	

a OR: odds ratio.

b BCI: Bayesian credible interval.

a *u* is scalar parameter representing the rate of decline of correlation with distance between points.

b *σ^2^* is the estimate of the geographic variability.

c DIC is the measure for the model fit. A smaller DIC indicates a better performance of the model.

The model performance improved considerably when the spatial random effect was introduced (DIC for Model 2 is much smaller than for Model 1). When the demographic covariates were introduced, the model performance improved again (Model 3). However, addition of reservoir host covariates, as well as environmental covariates did not have a significant influence on the model performance (Models 4 and 5) compared with Model 3.

## Discussion

We used epidemiologic data from the periodic epidemiologic survey carried out in 2004 by the Chinese Ministry of Health, and environmental data available from digitized maps and satellite images to determine demographic, reservoir host (infected buffaloes) and environmental indicators for the spatial distribution of *S. japonicum* infections in the Dongting Lake region of Hunan province. The overall prevalence among 47,139 study participants was 11.9% and 1.9% based on ELISA and Kato-Katz thick smear, respectively, and varied significantly between administrative villages. Age, sex, occupation, presence and prevalence of infected buffaloes, NDVI, distance to the lake and endemic type were all significant factors for infection with *S. japonicum* for both diagnostic methods. After taking into account spatial correlation, only the demographic factors and the presence of infected buffaloes could significantly explain the geographic variation of infection. Comparison of the different models revealed that demographic factors had the strongest influence on model performance compared to the reservoir host and environmental factors.

Sensitive diagnostic methods are a prerequisite for effective disease control. However, for schistosome infection there is currently no cheap, sensitive and specific test available [28]. In China, ELISA and Kato-Katz thick smear are routinely used for identification of infections during epidemiologic surveys. Yet, serology for diagnosis of schistosomiasis patients in areas where the infection is endemic does not discriminate between previous and current infection. Nonetheless, serology may be useful for the spatial targeting of control based on the following grounds: i) the ELISA test has a higher diagnostic sensitivity than the Kato-Katz thick smear that systematically underestimates *Schistosoma* infection prevalence [29], [30], consequently the chance to miss low intensity areas is smaller when the ELISA test is used; and ii) antibody responses to antigen can be mapped and hence areas where exposure occurs identified [31]. In this study we compared results derived from ELISA and Kato-Katz thick smear. The results of the non-spatial and spatial regression analyses were comparable suggesting that both outcome variables are useful for identification of spatial indicators of the risk. With regard to the use of ELISA results our assumption was that a positive antibody test would at least indicate prior exposure, and hence also past infections that had been successfully treated. This is of importance not only for identification of an exposed population but because it can also partly indicate the successful implementation of morbidity control in distinct areas.

Adult males engaged in herding, farming, boating and fishing were at a higher risk of infection compared to other study participants. This is consistent with findings from another cross-sectional study in Hunan province carried out in 16 villages [32]. In the Dongting Lake region, evidence suggests that men have more frequent, prolonged and extensive body surface water contact compared to women, which in turn explains their higher prevalence, as well as re-infection rate after treatment [33]. With regard to occupation as an indicator for infection risk, there are several other studies that confirm our findings [33], [34]. Fishermen have the most frequent water contact, followed by aquatic workers and farmers [34]. Among farmers, human infection is significantly associated with agricultural production in rice fields infested with the intermediated host snail, and with rates of schistosome infection in livestock, notably cattle and buffaloes. The human infection rate increases with the number, as well as the infection rate in animals [35]. Our findings confirm this pattern, with a positive association found between infection in humans and the presence, as well as the proportion, of infected buffaloes.

The investigated environmental factors, i.e. NDVI and endemic type of setting, were significantly associated with *S. japonicum* infection prevalence in the bivariate non-spatial analyses, which is consistent with previous findings [20], [32]. On the other hand, distance to the lake showed a positive association with infection risk. A common assumption is that with increased distance from the Dongting Lake, water contact becomes less frequent and consequently the infection risk decreases. Surprisingly, our results suggest the opposite and hence this result warrants further investigation. However, after taking into account spatial correlation, none of the environmental factors remained significant in the multivariate spatial models. Omission of spatial dependence in the data underestimates the standard error of model coefficients, and this could explain why in the study carried out by Yang and colleagues (2009) the type of endemic setting was significant, although they used a hierarchical structure in their model. Nonetheless, our results could indicate successful implementation of control taking place in the Dongting Lake region, as indicators such as NDVI and endemic type of setting were not significantly associated with the spatial risk of *S. japonicum* infections in humans. In fact, NDVI is used as a proxy for the presence of the *O. hupensis* intermediate host snail. Lack of spatial significance might suggest that in many places the intermediate host snails might have been successfully eliminated through environmental management (e.g. focal mollusciciding and environmental modification) on the one hand, while on the other hand other preventive methods and treatment have been successfully implemented. Although, based on our above-mentioned assumption, pre-control prevalences should be reflected by the ELISA data and therefore correlation between the ELISA results and environmental factors should be present even after control, it is conceivable that after some time antibodies against *S. japonicum* will disappear from the human circulation. Lack of correlation could hence indicate a change in endemicity after successful control.

To summarize, we used data available from the periodic epidemiologic survey carried out by the Chinese Ministry of Health in 2004 to spatially model the *S. japonicum* infection risk among humans in the Dongting Lake region of Hunan Province in China. Our results suggest that socio-demographic (i.e. age, sex) and economic factors (i.e. occupation) might be more important than environmental factors in explaining the spatial distribution of *S. japonicum* in this particular epidemiologic setting. In addition, the presence of infected livestock poses an increased risk. Importantly, our results highlight the focal distribution of *S. japonicum* in the area and its prevalence among particular occupational groups, including fishermen, farmers and boatmen who are in close contact with cercariae infested water. Integrated control efforts should hence be directed to those communities at risk and should include improved access to treatment and preventive measures, health education, focal mollusciciding, environmental modification, and improvement of sanitation and water-supply systems, as well the use of bovine vaccines [4], [5].
